# The Micro Hand S vs. da Vinci Surgical Robot-Assisted Surgery on Total Mesorectal Excision: Short-Term Outcomes Using Propensity Score Matching Analysis

**DOI:** 10.3389/fsurg.2021.656270

**Published:** 2021-05-11

**Authors:** Yijia Zeng, Guohui Wang, Zheng Li, Hao Lin, Shaihong Zhu, Bo Yi

**Affiliations:** Department of General Surgery, Third Xiangya Hospital, Central South University, Changsha, China

**Keywords:** total mesorectum excision, hospital costs, Micro Hand S surgical robot system, da Vinci surgical robotic system, robot-assisted surgery

## Abstract

**Objective:** To compare the operation mode and clinical short-term outcomes of the Micro Hand S and the da Vinci surgical robot, we chose total mesorectal excision (TME) as the standard procedure for its good reflection of robot-assisted surgery advantages.

**Methods:** We collected a total of 54 consecutive patients who underwent robot-assisted TME by two surgical robots from January 2016 to October 2020. We used propensity score matching (PSM) to create balanced cohorts of Micro Hand S group (*n* = 14) and da Vinci group (*n* = 14). Robotic installation and operation time, hospital and surgery costs, and intraoperative and postoperative clinical outcomes were compared.

**Results:** In terms of robotic installation time, the Micro Hand S robot took longer than the da Vinci robot (24.2 ± 9.4 min vs. 17.1 ± 5.1 min, *P* < 0.05). As for the costs, the Micro Hand S group had lower total hospital costs (87,040.1 ± 24,676.9 yuan vs. 125,292.3 ± 17,706.7 yuan, *P* < 0.05) and surgery costs (25,772.3 ± 4,117.0 yuan vs. 46,940.9 ± 10,199.7 yuan, *P* < 0.05) than the da Vinci group. There were no statistically significant differences (*P* > 0.05) in other indicators, including total operation time, robotic operation time, blood loss, time to first liquid diet, time of getting out of bed, and hospital stay.

**Conclusion:** The Micro Hand S enables patients with rectal cancer to enjoy lower medical costs of robotic surgery.

**Clinical Trial Registration:** ClinicalTrials.gov [NCT02752698]

## Introduction

The emergence of robot-assisted surgery is of epoch-making significance in the history of surgery. The equipment and instruments of surgical robots are constantly being innovated, enabling surgeons to perform increasingly complicated and subtle surgical procedures. Currently, the most widely used da Vinci surgical robot is characterized by 3D vision, tremor elimination, dexterity, and instruments with multiple degrees of freedom. These technological features are particularly advantageous when operating in narrow spaces within the body (1–3).

Surgical robots have been used in many types of operations due to their excellent operative performance. The total mesorectal excision (TME) for rectal cancer is very suitable for the use of surgical robots, because the entire surgery is completed in a confined space, which requires accurate and delicate anatomical operations (4–7). Many researchers believe that robotic surgery reduces the difficulty of the operation by making it easier to observe the layers of fascia and the pelvic nerves. Accordingly, studies have shown a better conversion rate and postoperative pathology with robotic surgery than with laparoscopic surgery (8–10). However, in the recent large ROLARR trial, it was found that there were no statistically significant differences between robotic and laparoscopic surgery in terms of conversion rate and other intraoperative and postoperative indicators (11). Although the outcomes are inconsistent with the hypothesis that the surgical robot should facilitate rectal cancer resection, the technological advantages of the robot are reflected in some specific procedures as the technical difficulty increases.

In this study, we select TME, which well reflects the advantages of robotic surgery, as a standard procedure to compare the operation mode and short-term effects of the two surgical robots so as to verify the safety and feasibility of the Micro Hand S surgical robot in the treatment of rectal cancer. The Micro Hand S is a novel surgical robot control system independently developed in China. It shares all the characteristics of robot-assisted surgery mentioned above but differs from the da Vinci surgical robot in operation mode, matched instruments, surgery cost, and other aspects (12–14). This study has been approved by the hospital ethics committee, and further information on the study can be found at ClinicalTrials.gov (NCT02752698).

## Materials and Methods

### Patients

We collected the clinical data of all patients who underwent robot-assisted TME using the Micro Hand S or the da Vinci surgical robot in the Third Xiangya Hospital, retrospectively from January 2016 to October 2020. The inclusion criteria were (1) 18–80 years old, male or female; (2) preoperative colonoscopic pathology that confirms or indicates a high suspicion of rectal cancer; (3) preoperative CT or MRI imaging examination that supports the diagnosis; (4) American Society of Anesthesiology (ASA) level I–III. Patients with T4 stage or obvious tumor invasion of surrounding tissues were excluded.

In total, 54 consecutive patients were included and divided into two groups, with 15 patients in the Micro Hand S group and 39 patients in the da Vinci group. None of the 54 patients received neoadjuvant treatment.

### Indicators

The preoperative characteristics of patients included age, gender, body mass index (BMI), ASA level, distance from tumor site to the anus, and clinical TNM stage (T1–T3). The intraoperative indicators included total operation time, robotic installation time, robotic operation time, and blood loss. Postoperative short-term outcomes included time to first liquid diet, time of getting out of bed, hospital stay, surgery costs, and total hospital costs.

Evaluation indicators of the excised specimens included lymph nodes harvested, positive rate of circumferential resection margin (CRM), distance to distal resection margin (DRM), distance to proximal resection margin, and macroscopical mesorectum integrity, which was divided into three degrees: complete, nearly complete, and incomplete. For CRM, excised specimens with a shortest distance ≤1 mm to the circumferential resection margin were defined as positive.

### Operative Procedures

The two surgeons were both professional gastrointestinal doctors with more than 15 years of experience in laparoscopic surgery and more than 5 years of experience in operating the da Vinci surgical robot. In addition, they had been operating the Micro Hand S surgical robot from the very first design stage to the clinical phase I stage and had completed more than 100 Micro Hand S robot-assisted surgeries. Therefore, they could be considered unaffected by the learning curve in both groups. All the surgeries were performed by these two surgeons, and the surgical team was the same.

#### The Micro Hand S Group

**Figure 1:**
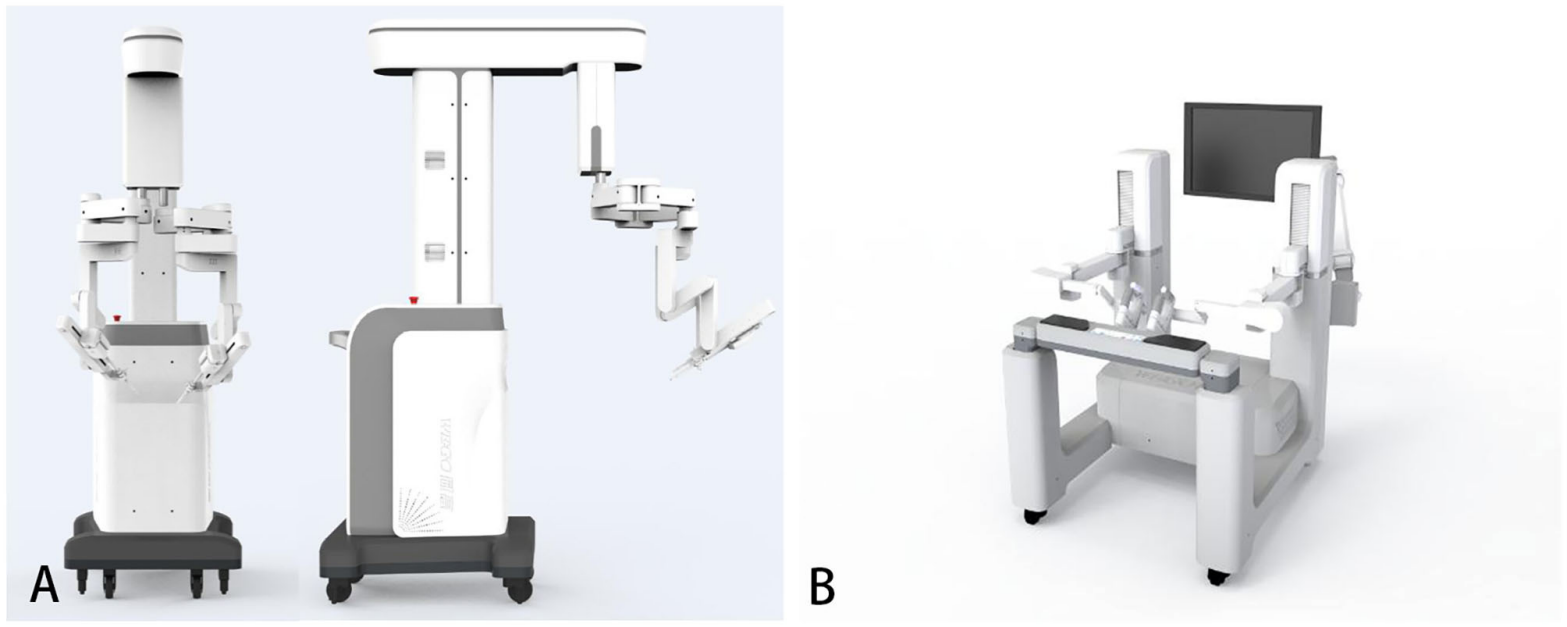
The Micro Hand S surgical robot system. **(A)** The patient's console. **(B)** The doctor's console (15).

**Figure 2:**
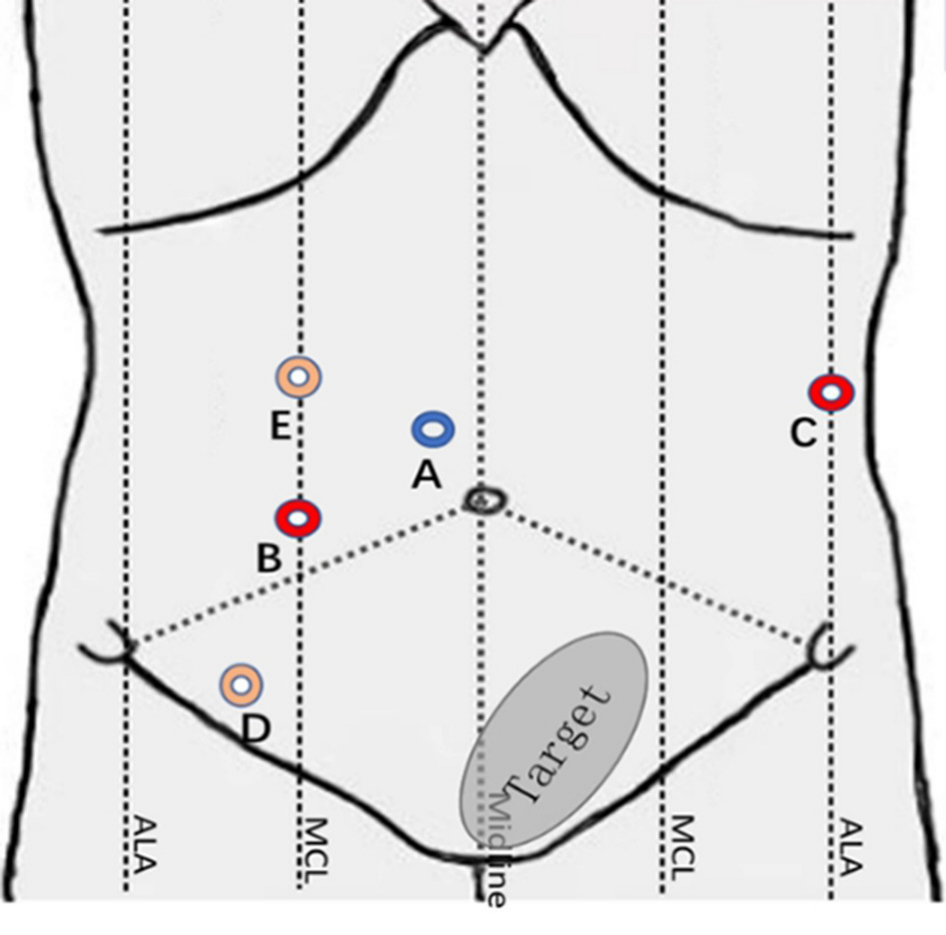
The sites of incision points of Micro Hand S robot-assisted TME.

The surgical robot is produced by Shandong Wego Surgical Robot Co., LTD. It is a master-slave robotic system as shown in Figure 1. Routine gastrointestinal preparation was begun 24 h before surgery. All patients were intubated under general anesthesia in the Trendelenburg position with 10°–15° right inclination. Then we established the pneumoperitoneum. Five sites of incision points were chosen as below (Figure 2): Point A was 3 cm upper the umbilicus to the right, left for the 3D camera (12 mm trocar); Point B was the intersection of right midclavicular line and umbilical plane, connected with the right operative arm (10 mm trocar). Point C was on the left anterior axillary line 5–8 cm below the costal margin, connected with the left operative arm (10 mm trocar). Point D was lateral to the right midclavicular line and 10 cm below Point B, chosen as an auxiliary incision for the assistant (12 mm trocar). Point E was on the upper side to the AB line, also an auxiliary incision for the assistant (5 mm trocar). The spacing of each puncture point was set to be 10 cm apart if possible in order to effectively avoid collision between the robotic arms and the assistants.

**Figure 3:**
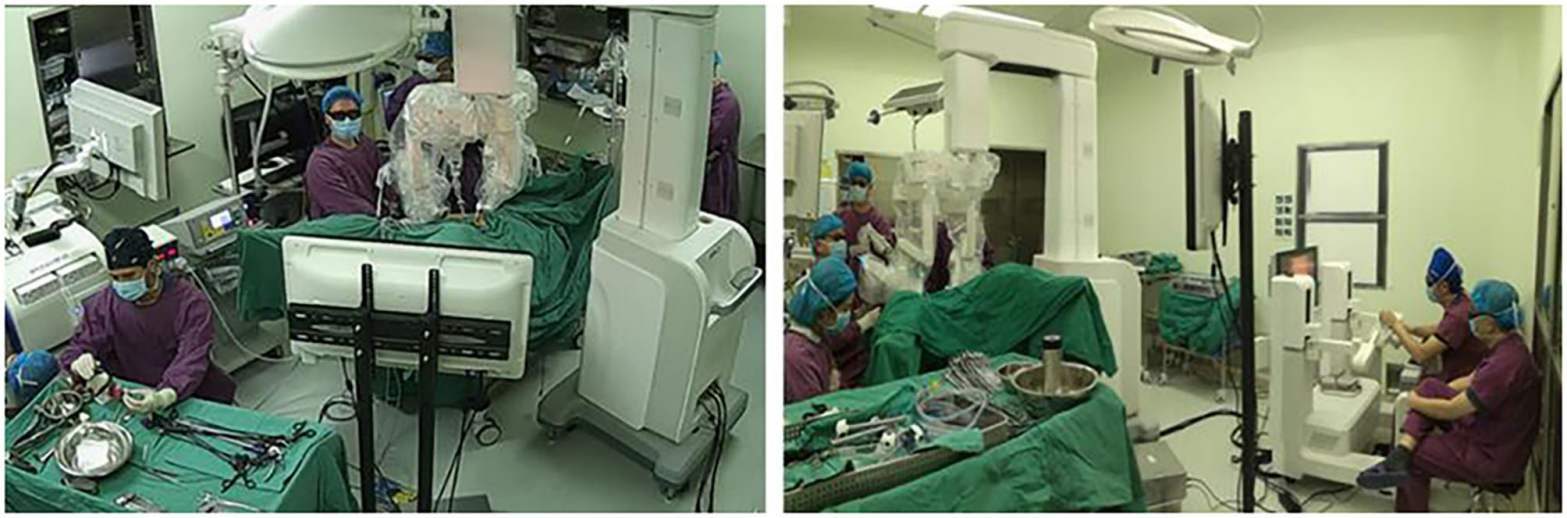
The operating room of the Micro Hand S robot-assisted TME.

The surgeon sat in front of the doctor's console and controlled the surgical instruments through the master hand (Figure 3). The first assistant was responsible for surgical area exposure, irrigation, use of vascular clips, and replacement of the matched instruments in the operative arms. The second assistant was responsible for holding the 3D laparoscopic lens and occasionally debugging. The surgeon can control two kinds of instruments at the same time. It is important to note that there was not a third operative arm to hold the camera when this study was conducted, so we needed a second assistant to do that.

**Figure 4:**
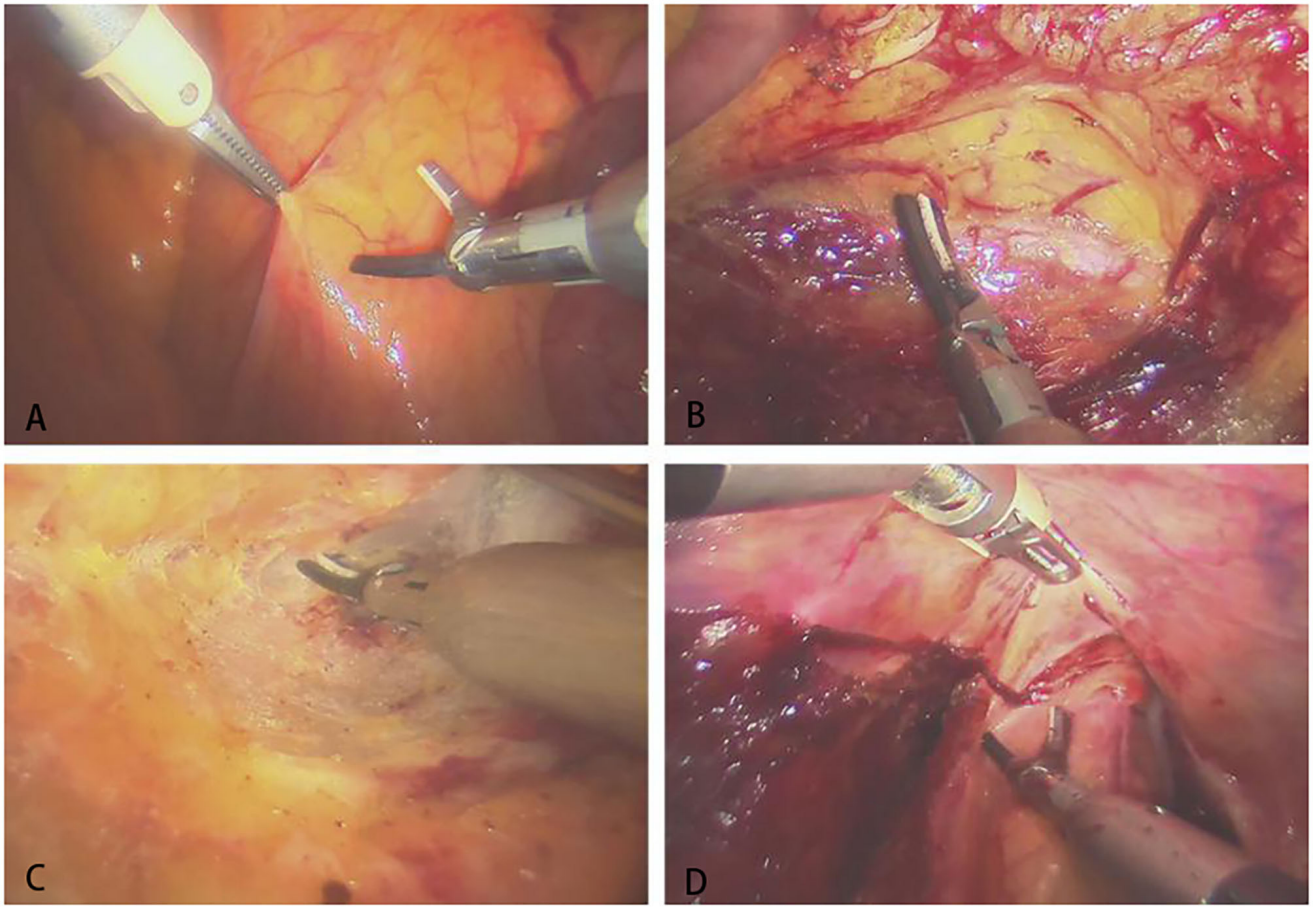
The intraoperative images of Micro Hand S robot-assisted TME. **(A)** Opening the posterior peritoneum; **(B)** dissociating the left ureter; **(C)** separating the presacral space; **(D)** separating the anterior rectal wall.

The assistant used laparoscopic tissue forceps to pull the descending colon and assist with surgical area exposure from medial to lateral. The surgeon manipulated the operative arms to dissect the descending colon and ligate the submesenteric vessels. Then the assistant pulled the sigmoid colon to the left for better exposure. The surgeon cut through the peritoneal reflection and dissociated the retrorectal space. The dissociation was performed meticulously between the presacral fascia and the fascia propria of the rectum, with great care taken to keep the Waldeyer's fascia and pelvic nerves and vessels intact. The prerectal space was dissociated with great care taken to preserve the Denonvilliers' fascia. When the surgeon separated the lateral mesorectum, the assistant pulled the tissues to maintain tension to better expose the avascular plane. After separation of the rectum, the tumor was located, and the rectum was transected with linear stapler. Finally, the operative arms were withdrawn, and the left incision was enlarged to 4–5 cm to remove the tumor. An end-to-end anastomosis between the sigmoid colon and the rectum was performed with a circular stapler (31 mm). The intraoperative images of Micro Hand S robot-assisted TME are shown in Figure 4.

#### The da Vinci Group

The da Vinci Si surgical robot is produced by Intuitive Surgical Co., LTD. The robot system has three parts: the doctor's console, the operative arms, and the imaging system. The patients underwent routine gastrointestinal preparation starting 24 h before surgery. They were intubated under general anesthesia in the Trendelenburg position with 10°–15° right inclination, and the pneumoperitoneum was established. The sites of trocars are the same.

All the surgical procedures were similar to the Micro Hand S group, except that there was one less assistant for holding the camera. The steps were basically the same as those mentioned in the Micro Hand S group, and all steps in both groups followed the principles of tumor-specific mesorectal excision. The specifics of each operation were at the discretion of the operating surgeon.

### Statistical Analysis

SPSS 23.0 software (SPSS Inc., Chicago) was used for data analysis. We performed the *t*-test of two independent samples for homogeneity of variance, and we performed the *t*'-test of two independent samples for inhomogeneity of variance. As the sample size was <40, Fisher's exact probability test was conducted for categorical data. The non-parametric rank sum test was used for ordered categorical data.

Propensity score matching (PSM) analysis was performed to minimize the selection bias. The concomitant variables were sex, age, BMI, ASA level, distance from tumor site to the anus, and clinical TNM stage. The two groups were matched according to the propensity scores using the nearest neighbor matching in a 1:1 ratio without replacement. After PSM, 14 patients each in the Micro Hand S and da Vinci groups were included for further analyses. Between the two groups, the baseline characteristics were well-balanced.

## Results

### Preoperative Characteristics of Patients

Totally in this study, 54 patients were enrolled, including 15 patients in the Micro Hand S group and 39 patients in the da Vinci group. After 1:1 PSM, the two groups showed no statistically significant difference (*P* > 0.05) in baseline characteristics (Table 1).

**Table 1:** Patient and tumor characteristics before and after PSM.

	**Before PSM**			**After PSM**		
	**Micro Hand S (*n* = 15)**	**da Vinci (*n* = 39)**	***p***	**Micro Hand S (*n* = 14)**	**da Vinci (*n* = 14)**	***p***
Sex, *n* (%)			0.074			0.706
Male	6 (40.0)	26 (66.7)		6 (42.9)	8 (57.1)	
Female	9 (60.0)	13 (33.3)		8 (57.1)	6 (42.9)	
Age (years)	60.6 (11.4)	58.4 (12.7)	0.554	61.6 (11.1)	66.1 (10.6)	0.282
BMI (kg/m^2^)	21.4 (2.7)	22.8 (2.9)	0.106	21.6 (2.7)	22.89 (3.1)	0.237
Distance from tumor site to the anus (cm)	8.6 (3.2)	7.2 (3.0)	0.130	8.9 (3.2)	8.4 (2.7)	0.645
ASA, *n* (%)			0.925			0.511
I	5 (33.3)	10 (25.6)		4 (28.6)	4 (28.6)	
II	6 (40.0)	21 (53.8)		6 (42.9)	9 (64.3)	
III	4 (26.7)	8 (20.5)		4 (28.6)	1 (7.1)	
cT, *n* (%)			0.145			0.769
1	0 (0.0)	3 (7.7)		0 (0.0)	0 (0.0)	
2	0 (0.0)	1 (2.6)		0 (0.0)	0 (0.0)	
3	6 (40.0)	19 (48.7)		6 (42.9)	7 (50.0)	
4	9 (60.0)	16 (41.0)		8 (57.1)	7 (50.0)	
cN, *n* (%)			0.007			0.104
0	14 (93.3)	21 (53.8)		13 (92.9)	8 (57.1)	
1	1 (6.7)	13 (33.3)		1 (7.1)	5 (35.7)	
2	0 (0.0)	5 (12.8)		0 (0.0)	1 (7.1)	
Clinical TNM, *n* (%)			0.783			0.769
I	0 (0.0)	4 (10.3)		0 (0.0)	0 (0.0)	
II	10 (66.7)	21 (53.8)		9 (64.3)	8 (57.1)	
III	5 (33.3)	14 (35.9)		5 (35.7)	6 (42.9)	

*Values are presented as mean (SD) or number with percentage. BMI, body mass index; ASA, American Society of Anesthesiologists*.

### Perioperative Short-Term Outcomes

None of the patients were converted to laparoscopic surgery. There was no perioperative death or anastomotic leak happened. All the perioperative indicators are shown in Table 2. The Micro Hand S robot took longer installation time than the da Vinci robot (24.2 ± 9.4 min vs. 17.1 ± 5.1 min, *P* < 0.05). As for the costs, the Micro Hand S group had lower costs than the da Vinci group in total hospital costs (87,040.1 ± 24,676.9 yuan vs. 125,292.3 ± 17,706.7 yuan, *P* < 0.05) and in surgery costs (25,772.3 ± 4,117.0 yuan vs. 46,940.9 ±10,199.7 yuan, *p* < 0.05). There were no statistically significant differences (*P* > 0.05) in other indicators, including total operation time, robotic operation time, blood loss, time of getting out of bed, time to first liquid diet, and hospital stay.

**Table 2:** The perioperative indicators and short-term outcomes of the patients in two groups.

	**Micro Hand S**	**da Vinci**	***p***
	**(*n* = 14)**	**(*n* = 14)**	
Total operation time (min)	260.6 (45.4)	256 (42.9)	0.783
Robotic installation time (min)	24.2 (9.4)	17.1 (5.1)	0.021^*^
Robotic operation time (min)	143.29 (36.0)	137.9 (27.4)	0.662
Blood loss (ml)	123.6 (60.2)	127.1 (105.5)	0.913
Anastomotic leak, *n* (%)	0/14 (0)	0/14 (0)	1.000
Perioperative death, *n* (%)	0/14 (0)	0/14 (0)	1.000
Hospital stay (*d*)	13.2 (6.3)	12.6 (4.2)	0.753
Time to first liquid diet (*d*)	2.4 (0.5)	2.4 (0.6)	0.746
Time of getting out of bed (*d*)	2.1 (0.3)	2.2 (0.4)	0.297
Total hospital costs (yuan)	87,040.1 (24,676.9)	125,292.3 (17,706.7)	0.000^**^
Surgery costs (yuan)	25,772.3 (4,117.0)	46,940.9 (10,199.7)	0.000^**^

*Values are presented as mean (SD)*.

* *P < 0.05,*

** *P < 0.001*.

### Evaluation of Pathological Specimen

No statistically significant difference was showed (*P* > 0.05) in number of lymph nodes harvested, positive rate of CRM, distance to DRM, distance to proximal resection margin, and macroscopical mesorectum integrity (Table 3).

**Table 3:** The evaluation of the excised specimens.

	**Micro Hand S (*n* = 14)**	**da Vinci (*n* = 14)**	***p***
Number of lymph nodes harvested (*n*)	15.8 (3.0)	15.5 (3.6)	0.776
Distance to PRM (cm)	8.2 (3.1)	9.5 (4.2)	0.919
Distance to DRM (cm)	2.3 (1.1)	2.3 (1.1)	0.374
Positive rate of CRM, *n* (%)	0/14 (0)	0/14 (0)	1.000
Macroscopical mesorectum integrity, *n* (%)			1.000
Complete	14 (100)	13 (92.9)	
Nearly complete	0 (0)	1 (7.1)	
Incomplete	0 (0)	0 (0)	

*Values are presented as mean (SD) or number with percentage. PRM, proximal resection margin; DRM, distal resection margin; CRM, circumferential resection margin*.

## Discussion

The Micro Hand S and the da Vinci surgical robot share some of the same features, such as 3D vision, nice dexterity, instruments with a high degree of freedom, and tremor filtering. However, they differ in the following aspects:

### Imaging Methods

The 3D image of the Micro Hand S surgical robot is widely open so that the surgeon and others can watch the screen of the doctor's console simultaneously wearing 3D glasses and exchange their thoughts while performing the procedure, sharing the same view. The 3D image of the da Vinci surgical robot is fully immersive, with the surgeon having exclusive access to the screen so that the surgeon can be highly focused.

### Action Mapping

The action mapping of Micro Hand S is adjustable with three options (1:3, 1:6, and 1:10), which means that the surgeon moving 3, 6, or 10 cm in the doctor's console will cause the operative arms to move 1 cm simultaneously. This design helps to adapt the operation for people with different operative experience. As the technical difficulty increases, they can choose to magnify the action mapping so that the operative arms move less for the same movement range in the doctor's console, resulting in a lower possibility of tissue injury. In contrast, the action mapping of the da Vinci surgical robot is fixed.

### Matched Instruments

Different from the da Vinci surgical robot, its end of the instruments adopts a combination of three degrees of freedom: rotation, swinging, and joint rotation. Both left and right operative arms can use energized instruments at the same time without influencing each other. For example, an ultrasonic knife can be manipulated by the left arm, and a bipolar electrocoagulation forceps can be manipulated by the right arm. A study found that the ultrasonic knife had a smaller thermal radiation range and was safer than monopolar electrocoagulation forceps in TME surgery, which could easily lead to mesenteric damage (16). In the da Vinci surgical robot, the end of the instruments adopts a combination of three degrees of freedom: rotation, swinging, and pitching. This combination is more dexterous and easier to adapt for experienced laparoscopic surgeons because the way it works is the same as that of a laparoscopic instrument. However, each instrument has a usage limitation, so it will be locked down and not able to be recognized after 10 uses.

In our study, in terms of total operation time and robot operation time, the difference between the two robots was not statistically significant. However, as for installation time, the Micro Hand S surgical robot took longer to recognize the chips during instrument installation, and sometimes it took more than once to successfully identify the instrument. Most studies believe that reasons like the extra installation and discharge step, the operator's insufficient operating experience, and the learning curve will affect robot-assisted surgery and prolong the operation time compared with laparoscopic surgery (17–20). However, there are also some studies showing that the operation time was comparable between robot-assisted and laparoscopic surgery (8, 9, 21). In the ROLARR trial, there was also no statistically significant difference between robot-assisted and laparoscopic surgery in terms of operation time (11). In this study, the two selected surgeons were both highly experienced and had years of cooperation with the surgical team. Therefore, the influence of the learning curve to the operation time can be disregarded.

Regarding hospital costs, the Micro Hand S group was significantly lower. In terms of the operative cost alone, for instance, the surgical instruments of the Micro Hand S surgical robot are about 1,000 yuan per set. There is no limitation on the number of times they could be used. The instruments of the da Vinci surgical robot cost about 2,000 yuan per set and can be used 10 times only. The other medical costs share unified pricing standards, including surgical consumables, perioperative examination, and therapeutic drugs. As for non-commercial health insurances, neither of the two types of robot-assisted surgery has been covered yet in China. It would be a fairly high price to pay comparing with laparoscopic surgery.

The intraoperative blood loss in the two groups was mostly 100–150 ml, including the bleeding when puncturing and sewing the skin. There was no significant statistical difference. Because using appropriately energized instruments in the two groups could achieve satisfactory hemostatic effects, unstoppable bleeding rarely occurred. As for comparing the robot-assisted surgery with laparoscopic surgery, some researchers found that the former led to more blood loss, which might be related to the instruments (17, 18). When separating the tissues and small vessels, a robotic energized instrument might need several energy releases to achieve effective hemostasis, whereas a laparoscopic energized instrument needs only one or two. This is inevitable because the transmission design of the robotic instrument sacrifices the maximum energy load to obtain better dexterity.

There was no statistically significant difference in postoperative complication incidence between the two groups, including severe complications such as intraoperative injury of peripheral tissues or organs and postoperative damage of urination function or sexual function. The two surgeons had rich experience in both robotic surgery and TME and were proficient in fascial layers, innervation, and vascularity. The pathology of the specimen was not significantly different between the two groups. In other researchers' comparative studies on robot-assisted and laparoscopic surgery, some concluded that there were no differences in postoperative pathological laboratory outcomes, local recurrence, 5-year survival rate, disease-free survival rate, and other indicators after long-term follow-up, which were consistent with the expected pathological results (11, 22, 23). However, some researchers found that the quality of pathological specimens and the prognosis in robotic surgery were better because experienced surgeons were more flexible and dexterous in performance when using a surgical robot (24, 25).

So far, neither Micro Hand S nor da Vinci surgical robots provide force feedback, instead providing only feedback from 3D images. In addition, the ends of instruments of the Micro Hand S surgical robot adopt a motion combination of rotation, swing, and joint rotation, sacrificing flexibility to allow higher energy loading for better hemostatic effect, which makes it unable to achieve as delicate operations as the da Vinci surgical robot could.

Finally, one obvious disadvantage of this study is that the sample size is too small. The phase I clinical study had spanned 5 years from 2014 to 2019 and we enrolled <150 patients. It was a hard time. Thankfully, in early 2021 a multi-center randomized controlled trial (RCT) covering 168 cases is about to be completed. We are now concentrating on this RCT and expecting more reliable clinical evidence. Furthermore, long-term prognosis of rectal cancer patients remains to be observed, and 5-year survival rate is an important indicator. We need longer follow-ups to enhance the reliability.

In conclusion, the Micro Hand S surgical robot for TME treatment of rectal cancer enables patients to enjoy lower medical costs of robotic surgery.

## Data Availability Statement

The original contributions presented in the study are included in the article/supplementary material, further inquiries can be directed to the corresponding author/s.

## Ethics Statement

The studies involving human participants were reviewed and approved by the Institutional Review Board of the Third Xiangya Hospital. The patients/participants provided their written informed consent to participate in this study.

## Author Contributions

SZ and BY contributed to the conception/design of the work. YZ and GW contributed to the acquisition of data. YZ, ZL, and HL contributed to the analysis/interpretation of data. YZ drafted the manuscript. YZ, SZ, GW, and BY revised the manuscript. All authors have approved the final version submitted and agreed to be accountable for all aspects of the work in ensuring that questions related to the accuracy or integrity of any part of the work are appropriately investigated and resolved.

## Conflict of Interest

The authors declare that the research was conducted in the absence of any commercial or financial relationships that could be construed as a potential conflict of interest.
